# Polymyxin B and low-dose hydrocortisone treatment in a patient with uroseptic shock in a rural health unit

**DOI:** 10.1093/omcr/omab109

**Published:** 2021-11-25

**Authors:** Toshio Arai, Yuichiro Mori, Saori Yoshizaki, Ryo Ando, Shunsuke Natori, Shun Morishita, Miyu Otani, Atsushi Numata, Hiroaki Osanai

**Affiliations:** 1 Department of Internal Medicine, Furano Kyokai Hospital, Hokkaido, Japan; 2 Department of Parasitology, National Institute of Infectious Disease, Tokyo, Japan; 3 General Education Center, Kyorin University Hospital, Tokyo, Japan; 4 Department of Gastroenterology, Teine Keijin-Kai Hospital, Hokkaido, Japan; 5 Department of Cardiology, Furano Kyokai Hospital, Hokkaido, Japan; 6 Department of Urology, Furano Kyokai Hospital, Hokkaido, Japan

## Abstract

Sepsis has a high mortality rate; thus, in the intensive care unit, early diagnosis and adjunctive treatments are crucial. However, generally, most patients with sepsis from rural area initially visit the emergency department at a rural hospital and are managed in general medical wards in Japan. Here we report on an 81-year-old Japanese female manifesting septic shock caused by the upper urinary tract infection of extended-spectrum beta-lactamase-producing *Escherichia coli* secondary to the left ureter obstruction by the urothelial carcinoma. Broad-spectrum antibiotics were administered. Although critical for the source control of infection, drainage of the ureteropelvic junction could not be performed immediately because of catecholamine-resistant hypotension. Hence, we administered polymyxin B-immobilized fiber column direct hemoperfusion, followed by low-dose hydrocortisone administration. After 8 hours of infusion, she recovered from the septic shock and successfully underwent emergency percutaneous nephrostomy. This presented strategy may provide a new resolution of catecholamine-resistant patients in urosepsis.

## INTRODUCTION

Urosepsis caused by urinary tract infection (UTI) accounts for 20%–30% of sepsis cases, and uroseptic shock is a disease with high mortality, mainly caused by obstructive uropathy [1]. Polymyxin B-immobilized fiber column direct hemoperfusion (PMX-DHP) is a device that purifies blood by neutralizing circulating endotoxins, thereby inhibiting the progression of the septic cascade mediated by lipopolysaccharide, a core lipid portion of the gram-negative bacterial wall [2]. Studies have demonstrated that PMX-DHP reduces hospital mortality and the length of intensive care unit stay in patients with acute-phase septic shock control [2, 3]. However, the long term effect of PMX-DHP on survival rate has been insignificant in the ABDOMIX and EUPHRATES studies, despite an improvement in mean arterial pressure elevation [4, 5]. Glucocorticoids increase the sensitivity of alpha- and beta-adrenaline receptors. An adjunctive low-dose steroid has been reported to significantly shorten the time to recover from septic shock [6]. Various effects of glucocorticoids, including immune-modulating properties through interaction with NF-κB and elevation of blood glucose levels by stimulating glycogenesis in the liver and peripheral tissues, are favorable against septic shock [7].

In Furano city, a rural area in the northern part of Japan, there are no facilities such as helicopters to transfer patients to an intensive care unit of an urban hospital after sunset. Such patients need to be treated with available medical resources. In this case report, we present a case of uroseptic shock caused by the obstructive pyelonephritis, that was successfully treated at a rural hospital with a combination of PMX and a low-dose steroid.

## CASE REPORT

**Figure 1:**
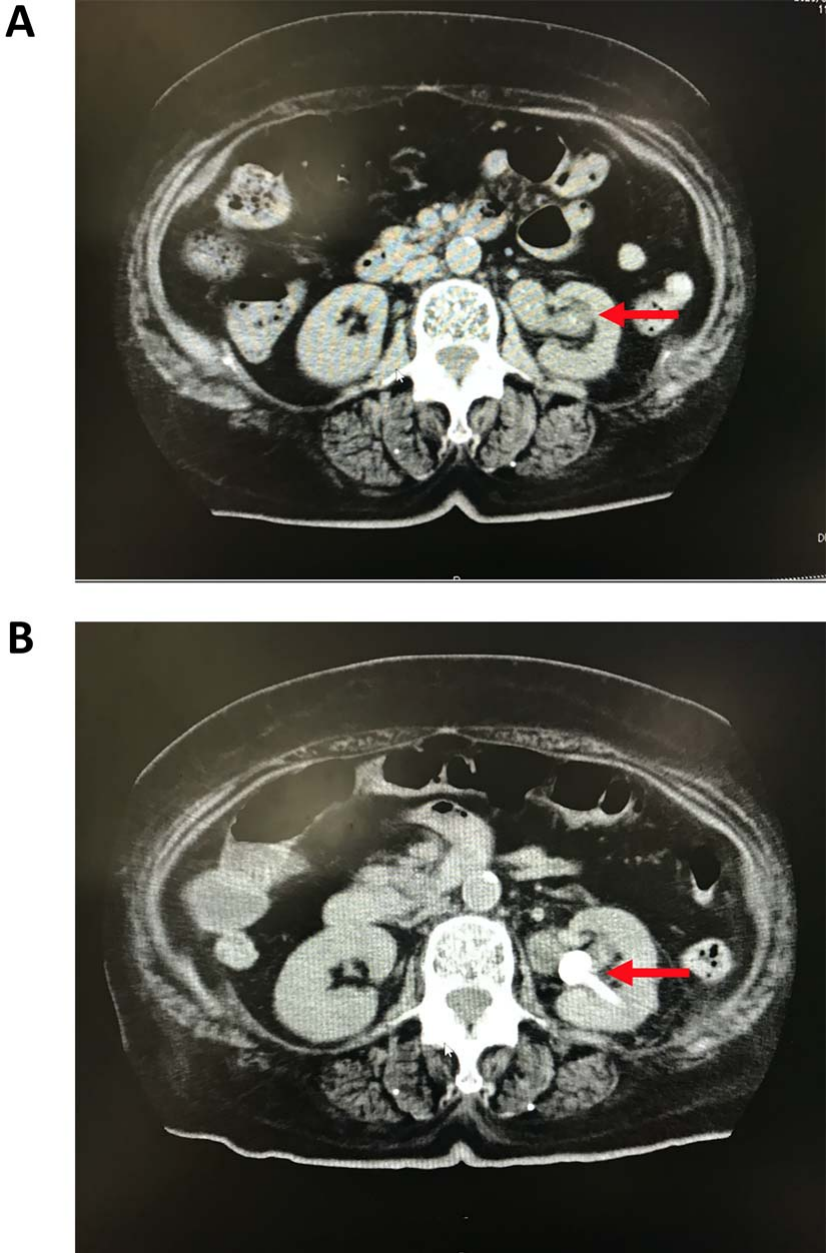
Computed tomography image of percutaneous nephrostomy. (a) Debris present in the left dilated renal pelvis (arrow). (b) The percutaneous nephrostomy tube properly placed in the left kidney (arrow).

An 81-year-old Japanese woman with twice histories of UTI presented to our emergency department with fever, anorexia and immobility around 4 pm. Her initial physical findings presented clear consciousness, body temperature of 37.5°C that continued since 3 days ago, upper extremity blood pressure (BP) of 68/36 mm Hg, heart rate (HR) of 84 bpm with irregular rhythm, respiratory rate of 24 cycles per minute, and oxygen saturation of 95% with 6 L/min of 100% oxygen administration via a Venturi mask. Physical examination revealed tenderness in the left costovertebral angle. The urine was white and cloudy in appearance and the leukocyte esterase test was positive. Microscopic examination revealed more than 50/hpf of white blood cells (WBC). Blood test revealed significantly increased levels of inflammatory markers, such as WBC, 27.1 × 10^3^ cells/μl with 93.8% of polymorphonuclear leukocytes; blood urea nitrogen, 31.2 of mg/dl; creatinine, 2.7 mg/dl; C-reactive protein, 26.7 mg/dl; glucose, 70 mg/dl; lactate, 3.3 m mol/L; and endotoxins, 2000 pg/ml. The arterial blood gas test revealed a pH of 7.39, pCO_2_ of 33 mm Hg, and pO_2_ of 80.9 mm Hg. Electrocardiogram showed atrial fibrillation. Noncontrast computed tomography imaging revealed a left ureteropelvic junction (UPJ) obstruction by a mass containing low-density fluid, which was diagnosed as an abscess by the radiologist (Fig. 1a). She was diagnosed with uroseptic shock secondary to the obstructive pyelonephritis with a Sequential Organ Failure Assessment score of 3 points.

**Figure 2:**
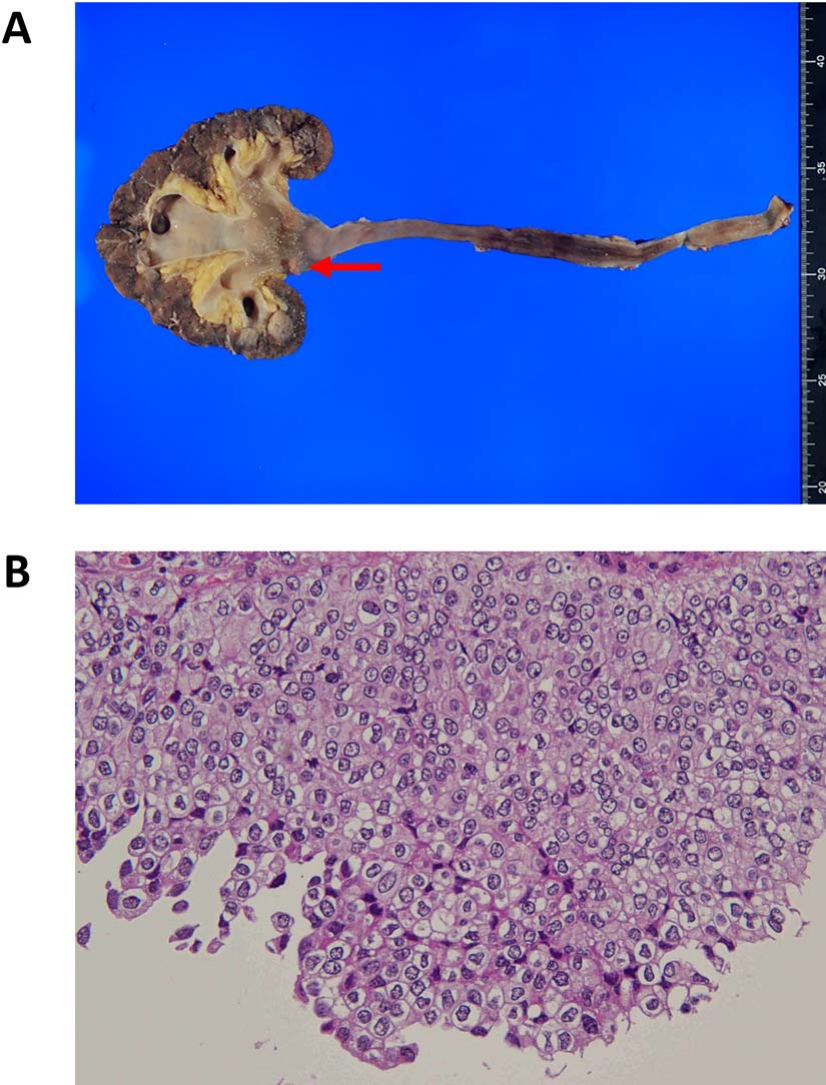
Gross and microscopic pathology. (a) Gross examination shows the urothelial carcinoma of the left renal pelvic (arrow). (b) Histopathologic examination shows high-grade noninvasive papillary urothelial carcinoma of the renal pelvis, hematoxylin and eosin, ×40.

The initial treatment comprising administration of intravenous fluids (2000 ml), intravenous norepinephrine of up to 0.3 μg/kg/min, and carbapenem 1.0 g was started; however, the shock vitals persisted. Thus, the initial source control by percutaneous nephrostomy was not performed due to the hypotension. To further account for hypotension and sepsis simultaneously, PMX-DHP was initiated and intravenous hydrocortisone of 240 mg was administered. The BP, HR and mean arterial pressure recovered to 125/80 mm Hg, 95 mm Hg and 80 bpm, respectively, in 8 hours. Then, the percutaneous nephrostomy was performed and the abscess at the UPJ was successfully drained (Fig. 1b). Urine and blood culture results reported on Day 4 revealed the presence of extended-spectrum beta-lactamase (ESBL)-producing *Escherichia coli* and therefore, the initial treatment of carbapenem was continued. The patient completely recovered in 2 weeks. She finally underwent total left nephroureterectomy on Day 65 with uneventful recovery. The pathological diagnosis revealed a high-grade noninvasive papillary urothelial carcinoma (Fig. 2a and b).

## DISCUSSION

Source control of urosepsis is the most crucial step. Treatment of urosepsis associated with obstruction and abscesses requires drainage [1]. Physiological changes may occur due to the prone position maintained during the drainage, such as increased intraperitoneal pressure and reduced venous return, which can potentially induce complications such as hypotension, arrhythmia, and syncope [8]. Accordingly, optimal BP must be maintained before such a procedure. In our case, UPJ drainage in the prone position was too invasive with poorly controlled BP after the initiation of norepinephrine. Hence, PMX-DHP along with the administration of hydrocortisone was selected in advance to the drainage. The effect of PMX on septic shock patients with different endotoxin levels is currently being investigated in a clinical study, the results of which are expected to come in 2022 (NCT03901807). It is known that norepinephrine, which is a potent alpha-adrenergic agonist with minimal beta-adrenergic agonist effects [9], is the first line of treatment for septic shock hypotension; however, our patient showed catecholamine-resistant hypotension with hypoglycemia and lactic acidosis. The use of corticosteroids and PMX improved hypoglycemia and hypotension simultaneously before using vasopressin.

ESBL-producing *E. coli* are resistant to numerous β-lactams, including expanded-spectrum cephalosporins. Major risk factors for colonization or infection with ESBL-producing organisms are antibiotic exposure, prolonged hospital stays, and admission in an institution with high rates of third-generation cephalosporin use [1]. In the present case, the patient had a medical history of UTI treated with cefcapene pivoxil, a third-generation cephalosporin, and it was suspected that the bacteria were already resistant to those antibiotics; thus, we chose an empirical treatment with carbapenem.

Limited or delayed access to proper medical care is a severe confounder of mortality in rural areas [10]. Even the critically-ill patients in rural areas reach hospital after some delay due to the distance. Furthermore, patient transfer was not an option in our case. A harmonized and efficient initial management, including correct diagnosis, vital sign control, empirical antimicrobial treatment and additional adjunctive treatment, is crucial in such cases.

In conclusion, we report a case of urosepsis in a rural area hospital that achieved optimal initial management, which included norepinephrine with hydrocortisone, PMX-DHP, and empirical antibiotics, ultimately resulting in good prognosis.
